# Two Cases Reported by Hahnemann to Bœnninghausen

**Published:** 1894-05

**Authors:** Wm. Bœricke


### TWO CASES REPORTED BY HAHNEMANN TO BŒNNINGHAUSEN.

### Case I.

O., actor, æt. thirty-three, married. January 14th, 1843.—Has been frequently troubled with an affection of the throat for several years past; has a new attack which has lasted already for a month. When swallowing saliva he feels a stinging sensation, tight and sore feeling.

When the throat is not affected he suffers with a fissure in the anus, painfully smarting; the anus is then swollen, inflamed, and narrower than usual; the expulsion of the fæces is very difficult under these circumstances, and is accompanied by the protrusion of hemorrhoids. Bellad.$^{100}$, dissolved in seven teaspoonfuls of water, one tablespoonful to be mixed in a tumblerful of water; one teaspoonful of this last mixture at a dose.

January 15th.—The sore throat was worse in the evening.

January 16th.—The sore throat had disappeared, but the affection of the anus had returned. Painful stool in the morning.

He confessed that he had had a chancre eight years ago, the removal of which by cauterization had been followed by the above-named symptoms. On the 16th he took Merc-viv., one pellet, prepared and taken as above.

January 20th.—Sore throat had almost gone. Anus improved; yet feels some soreness after stools; pulsations, swelling, and inflammation had disappeared. The narrowing was less. Merc-viv., one pellet, of the second higher dynamization, prepared in the same way as before, and taken in the morning. (It is not stated whether the Mercury was taken once or twice; generally only once in the morning.)

January 25th.—Throat almost well, but smarting pain and violent stitches in the anus; violent pain in the anus after stool; some narrowing and heat.

January 30th.—Last dose (one teaspoonful) in the afternoon.

On the 28th the anus was better, the sore throat had returned; the smarting in the throat was pretty violent. One pellet of Sugar of Milk, dissolved as before, and taken for seven days, one teaspoonful a day.

February 7th.—Considerable ulcerative pain in the throat. Colic; good stools, but several in succession, with great thirst. The anus is perfectly well. Sulphur, one pellet of the second potency in seven teaspoonfuls, as before.

February 13th.—Had an ulcerative pain in the throat, especially when swallowing saliva, which he now secretes in abundance, especially on the 11th and 12th.

The anus has become a little narrower, especially since yesterday. Smelled of Mercury, and took Merc-viv., second highest potency, one pellet to be dissolved in seven teaspoonfuls of water, to which was added half a teaspoonful of brandy; mix one teaspoonful in a tumblerful of water, and take one teaspoonful, as before.

February 20th.—The throat has been better since the 18th; great pains in the anus; stool is painful when passing it; thirst decreased. Sac-lac.

March 3d.—No sore throat. When passing stools, an empty hemorrhoidal tumor makes its appearance, with itching of the part (formerly with burning and smarting).

Smelling Ac-nitr. and Sugar of Milk, in seven tablespoonfuls, etc.

March 20th.—The pain after stool has almost gone; yesterday he passed some blood with the stool (old symptom). The throat is sound; there is a slight sensation when drinking cold liquids. Smelling of Ac-nitr. (Smelling is performed by opening a little vial containing one-half ounce of diluted Alcohol or Brandy, and smelling for one or two moments of a pellet which has been dissolved in it.) Remained well ever since.

### Case II.

Julia M., country girl, æt. fourteen, has not yet menstruated. September, 1842. Sleeping in the sun a month ago. Four days after having slept in the sun she imagined she saw a wolf; six days after this she felt as if she had been knocked in the head. She became delirious, frantic, wept a good deal, sometimes breathed with difficulty, spit up white mucus, was unable to say what she felt.

She took Bellad. in seven teaspoonfuls of water; shake the solution; mix one tablespoonful of it with a tumblerful of water; take one tablespoonful as a dose.

September 16th.—More calm; was able to blow her nose, which she was unable to do in her frenzy; she is yet delirious, but does not make so many gesticulations; wept a good deal the night previous; stool normal; sleep pretty good; is yet restless, but was a good deal more so previous to taking Bellad. The capillaries of the eye are considerably injected. Appears to have a pain in the nape of the neck. To pour one teaspoonful from the tumbler in which one tablespoonful had been mixed into a second glass of water, and take every morning from two to four teaspoonfuls of the second mixture (increasing the dose by one teaspoonful every morning).

September 20th.—Much better, speaks more rationally, wants to do something, calls me by my name, and wants to kiss a lady who is present. This was the commencement of a sort of sensualism which now manifested itself. She is easily irritated, fault-finding, sleeps well, weeps frequently, gets angry about trifles, eats more than usual; when she is in her senses likes to play, but like little children. Bellad., one pellet, to be dissolved in seven tablespoonfuls, one tablespoonful of which to be mixed in another tumblerful of water, taking one teaspoonful a day, early in the morning.

September 28th.—Considerable irritation on the 22d, 23d, 24th, day and night; great lasciviousness in manner and words, raises her frocks and wants to touch the genitals of other persons; gets angry easily and strikes everybody. Hyosc.$^{100}$, prepared as the Bellad., one teaspoonful as a dose.

October 5th.—Had not been willing to eat anything for five days past; complains of colic; is less angry and lascivious, more rational. Stool very soft, itching over the whole body, especially in the region of the genital organs. Sleep sound. Sac-lact., for seven days, one teaspoonful as above.

October 10th.—On the 7th she had a violent fit of anger, wanted to strike everybody. Next day fit of fear and tendency to start, as at commencement of her disease (fear of an imaginary wolf); she imagines she is going to be burnt. Since then she had become calm, and had talked rationally and with perfect propriety for the last two days. Sac-lact., etc.

October 14th.—Feels well and is rational.

October 18th.—The same; has sometimes a little headache; disposition to sleep in daytime; less cheerful. Sulphur, one pellet in three successive tumblers, one teaspoonful early in the morning.

October 22d.—Feels very well, has very little headache. Sulphur, next lower potency, in two tumblers. She used Sulphur occasionally until November, and remained a healthy, sensible, lovely girl.

